# The effects of antithrombotic therapy on head trauma and its management

**DOI:** 10.1038/s41598-021-00091-2

**Published:** 2021-10-14

**Authors:** Takao Koiso, Masayuki Goto, Toshitsugu Terakado, Yoji Komatsu, Yuji Matsumaru, Eichi Ishikawa

**Affiliations:** 1 Department of Neurosurgery, Hitachi General Hospital, Jonancho 2-1-1, Hitachi, Ibaraki 317-077 Japan; 2 Department of Neurosurgery, Faculty of Medicine, University of Tsukuba, Amakubo 2-1-1, Tsukuba, Ibaraki 305-8576 Japan

**Keywords:** Drug safety, Trauma, Stroke

## Abstract

The number of patients with traumatic intracranial hemorrhage (tICH) that are taking antithrombotics (ATs), antiplatelets (APs) and/or anticoagulants (ACs), has increased, but the influence of it for outcome remains unclear. This study aimed to evaluate an influence of AT for tICH. We retrospectively reviewed all patients with tICH treated between 2012 and 2019, and analyzed demographics, neurological status, clinical course, radiological findings, and outcome data. A total of 393 patients with tICH were included; 117 were on AT therapy (group A) and 276 were not (group B). Fifty-one (43.6%) and 159 (57.6%) patients in groups A and B, respectively, exhibited mRS of 0–2 at discharge (p = 0.0113). Mortality at 30 days was significantly higher in group A than in group B (25.6% vs 16.3%, p = 0.0356). Multivariate analysis revealed that higher age (OR 32.7, p < 0.0001), female gender (OR 0.56, p = 0.0285), pre-injury vitamin K antagonist (VKA; OR 0.42, p = 0.0297), and hematoma enlargement (OR 0.27, p < 0.0001) were associated with unfavorable outcome. AP and direct oral anticoagulant were not. Hematoma enlargement was significantly higher in AC-users than in non-users. Pre-injury VKA was at high risk of poor prognosis for patients with tICH. To improve outcomes, the management of VKA seems to be important.

## Introduction

Intracranial hematoma and its expansion are associated with unfavorable outcome of patients with head trauma^1–3^. As the Japanese population ages, the number of patient with traumatic intracranial hemorrhage (tICH) that are taking antithrombotic agents (AT), antiplatelet (AP) and/or anticoagulant (AC) therapy, is increasing. However, there is a discrepancy in the influence of pre-injury AT on outcomes in patients with head trauma. Furthermore, there remains no strong consensus on which patients should receive antithrombotic reversal agents, due to increased risk of thromboembolic events^4^. In addition, to our knowledge, there are no reports about the effect of resumption of AT after head trauma and the timing of resumption. Hence, use of reversal agent, resumption of AT, timing of resumption, and rebleeding risk of resumption after tICH are clinical conundrums. We hypothesize that the patients with tICH on AT therapy would be more likely to have unfavorable outcome than those without AT and reversal agent would be reduced hematoma expansion but increased thromboembolic events. The aim of this study was to evaluate the clinical factors, especially pre-injury AT, affected outcomes in patients with tICH. In addition, to assess the effects of use of reversal agent, resumption of AT, optimal time to restart AT, and rebleeding risk of resumption, the patients with AT in which thromboembolic events occurred after using reversal agents or rebleeding occurred after AT were restarted were extracted.

## Methods

### Ethical approval

All procedures performed in this study was approved by the local ethics committee of the Hitachi General Hospital (Research Ethics Committee of Corporate Hospital Group, Hitachi, Ltd: #2019-71), and complied with the conditions laid out by the Declaration of Helsinki. The opt-out consent was used for patients and the requirement to obtain informed consent was waived by Research Ethics Committee of Corporate Hospital Group, Hitachi.

### Patient population

This study was conducted at the Hitachi General Hospital, a critical care medical center. We retrospectively reviewed the medical records of all patients with head trauma who were hospitalized for treatment during the 7-year period between April 2012 and March 2019. Head trauma was defined as all damages of soft tissue of the head, skull, and/or intracranial regions. Patients who were aged under 16 years old, with chronic subdural hematoma, without intracranial hematomas, with spontaneous intraparenchymal hemorrhage (IPH), and whose modified Rankin Scale (mRS) was ≥ 3 before the trauma were excluded.

The following data were collected from medical records: age, sex, main type of tICH, Marshall classification, emergency surgery, IPH, international normalized ratio (INR), activated partial thromboplastin (APTT), extracranial injury, hematoma enlargement within 72 h, mRS at discharge, mortality within 30 days, and neurological death. Main type of tICH was defined if it seemed to be the major factor affecting symptoms.

### Imaging protocol

All computed tomography (CT) scans were performed using a SCENARIA CT scanner (Hitachi Medical Corporation, Hitachi, Japan) with a 2.5-mm slice thickness. All radiologists blinded and did not know the object of this study. All head trauma patients that were admitted to Hitachi General Hospital underwent an initial head CT scan within 60 min of arrival and a 2nd CT scan was performed within 24 h of admission if intracranial hemorrhage was identified on the initial CT scan. The existence and enlargement of intracranial hematomas were evaluated by the first author. IPH volume was calculated using the “ABC/2” formula^5^. “Enlargement” of IPH was defined as when the hematoma volume increased by more than 25%. In the case of epidural hematomas, subdural hematomas, or subarachnoid hemorrhage, “enlargement” was judged by authors based on the thickness of the thickest part. Rebleeding was defined when hematoma enlargement was identified in the patients, in which hemostasis was confirmed at least once in repeated CT.

### Reversal agents

All patients with tICH were checked international normalized ratio (INR) on arrival and within 24 h after administration if reversal agent was used. In Japan, KCENTRA (CSL Behring, King of Prussia, PA, USA) is the only approved 4-factor prothrombin complex concentrate (4F-PCC) by Japan’s pharmaceutical affairs since March 2017 as the urgent reversal of acquired coagulation factor deficiency induced by Vitamin K antagonist (VKA) therapy. Before approval of this drug, patients on VKA were injected 5 or 10 mg vitamin K2 (Vit. K2) intravenously. After approval, the indicated dose of 4F-PCC was administered in addition to Vit. K2; patients with an INR of 2–4 was 25 IU/kg, INR of 4–6 was 35 IU/kg, and INR more than 6 was 50 IU/kg. All patients on AP agents did not receive platelet transfusions or l-deamino-8-d-arginine vasopressin.

### Clinical outcomes

The primary endpoint was a favorable outcome, defined as mRS 0–2 at discharge. The secondary endpoints were enlargement of the intracranial hematoma within 72 h, all-cause mortality within 30 days, and in-hospital neurological death. Neurological death was defined as death caused by any intracranial disease that was identified by neurosurgeons.

To investigate the optimal time to restart AT treatment, we evaluated the cases in which rebleeding occurred after antithrombotic drug treatment was restarted or in-hospital thromboembolic events, which include ischemic stroke, myocardial infarction (MI), pulmonary embolism (PE), and venous thromboembolism (VTE), occurred, and reviewed past literatures.

### Statistical analysis

To determine the features of clinical characteristics of AT users, we compared clinical factors between the patients that were and were not treated with such drugs. To determine the clinical factors that influenced clinical outcome of patients with tICH, univariate and multivariate logistic regression analysis was performed. The variables that were statistically significant by univariate analysis were included in multivariate analysis.

Summary statistics for the examined variables are presented (frequencies and percentages for categorical data and medians and interquartile ranges for continuous data). Fisher’s exact test was used for analyzing categorical data, and the Wilcoxon rank sum test was used for analyzing continuous data.

All comparisons were planned, and all tests were 2-sided. P-values of < 0.05 were considered to be statistically significant. All statistical analyses were performed using JMP (Japanese version 12 for Windows; SAS Institute Inc., Cary, NC, USA).

## Results

### Study selection and characteristics

**Figure 1:**
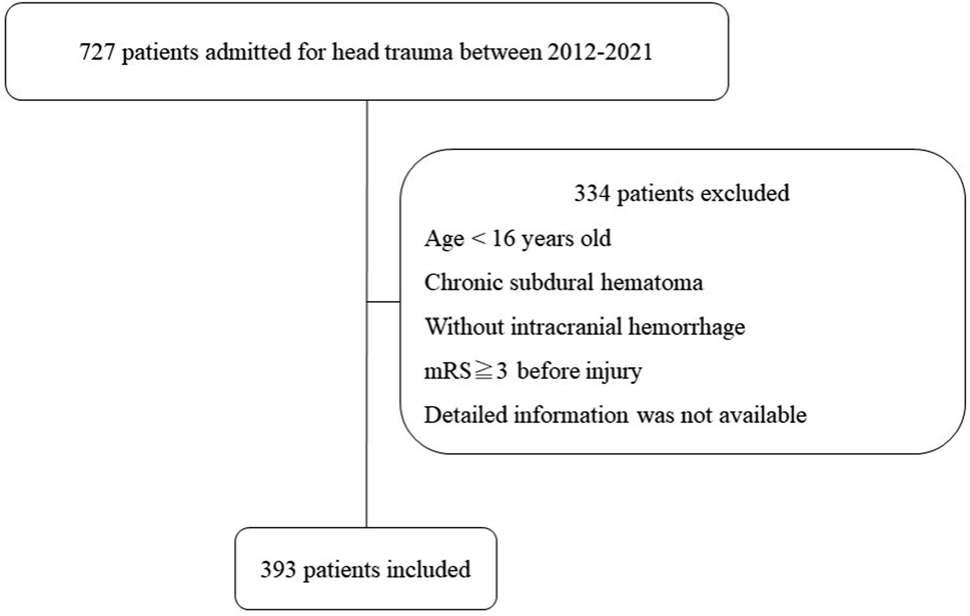
Schematic drawing of patient selection method.

A total of 727 consecutive patients with head trauma were identified during 7-year period. After excluding 50 patients who were aged under 16 years old, 206 patients with chronic subdural hematomas, 62 patients without intracranial hematomas, 4 patients with spontaneous intraparenchymal hemorrhage (IPH), 9 patients whose modified Rankin Scale (mRS) was ≥ 3 before the trauma, and 3 patients for whom detailed information was not available, 393 patients with tICH were included in this study (Fig. 1). Of the 393 patients, 46 did not undergo a 2nd CT scan before emergency surgery. Another 20 further patients did not undergo a 2nd CT scan because they died or had very small hematomas. Thus, 327 patients, 44 of whom with AC, were performed repeated CT scan at least once within 24 h. They were included in the calculation of the ratio of hematoma enlargement within 72 h. In addition, totally 359 patients were performed CT or MRI at least once during hospitalization. The incidence of ischemic stroke or rebleeding were evaluated in them.

Table 1 summarizes the clinical characteristics of the whole study population and the two groups; i.e., the patients that were (group A, 117 patients) and were not (group B, 276 patients) treated with AT. In group A, 62 patients (53.0%) were using AP alone, 38 patients (32.5%) were using AC alone, and 17 patients (14.5%) were using both types of drugs. Aspirin was the most frequently used antiplatelet medication (49 patients, 62.0%), followed by clopidogrel (26, 32.9%), cilostazol (12, 15.2%), and others (7, 8.9%). In these 79 patients, dual antiplatelet therapy (DAPT) was performed in 24 patients (30.4%) and triple antiplatelet therapy (TAPT) in 1 patient (1.3%). On the other hand, VKA was more frequently used anticoagulant medication (42 patients, 76.4%), followed by direct oral anticoagulant (DOAC) (13, 23.6%). The most common reason for AP use was a cardiovascular event [35/66 (43.2%); i.e., myocardial infarction] followed by a cerebrovascular event [25/66 (30.9%)]. The most common reasons for using AC were atrial fibrillation or cardiac valve surgery [47/53 (85.5%)]. The reasons for AT use were unknown in 13 patients who were using AP and 2 patients who were using AC. The median age of group A was significantly higher than that of group B (79.0 vs. 71.0, p < 0.0001). IPH was found in 42 (35.9%) patients in group A and 133 (48.2%) patients in group B (p = 0.0268). Eighteen (15.4%) patients in group A and 79 (28.6%) patients in group B had extracranial injuries (p = 0.0049).

**Table 1:** Summary of demographic and clinical characteristics of 393 patients with traumatic intracranial hemorrhage.

	Total	Antithrombotic drug treatment		p-value
		Yes (group A)	No (group B)	
No. of patients	393	117	276	
**Age, years**				
Median	75.0	79.0	71.0	< 0.0001
IQR	64.0–81.0	75.5–83.5	60.3–80.0	
**Sex**				
Female	129 (32.8%)	46 (39.3%)	83 (30.1%)	0.0791
Male	264 (67.2%)	71 (70.7%)	193 (69.9%)	
**Main type of intracranial hemorrahge**				
SAH	205 (52.2%)	36 (30.8%)	169 (61.2%)	
IPH	90 (22.9%)	23 (19.7%)	67 (24.3%)	
SDH	67 (17.0%)	54 (46.2%)	13 (4.7%)	
EDH	31 (7.9%)	4 (3.4%)	27 (9.8%)	
**Marshall CT classification**				
DI I	0	0	0	
DI II	296 (75.3%)	81 (69.2%)	215 (77.9%)	
DI III	14 (3.6%)	3 (2.6%)	11 (4.0%)	
DI IV	4 (1.0%)	1 (0.9%)	3 (1.1%)	
DI V	0	0	0	
DI VI	79 (20.1%)	32 (27.4%)	47 (17.0%)	
Emergency operation for head trauma	85 (21.6%)	29 (24.8%)	56 (20.3%)	0.3491
Existence of IPH	175 (44.5%)	42 (35.9%)	133 (48.2%)	0.0268
**PT-INR**				
Median	0.98	1.07	0.97	< 0.0001
IQR	0.92–1.07	0.95–2.00	0.91–1.03	
**APTT**				
Median	29.3	30.9	28.3	< 0.0001
IQR	26.8–31.9	28.1–34.9	26.3–31.0	
Extracranial injury	97 (24.7%)	18 (15.4%)	79 (28.6%)	0.0049
AND	27 (6.9%)	11 (9.4%)	16 (5.8%)	0.1977
**Enlargement of intracranial hematoma < 72 h**				
Yes	108/327 (33.0%)	30/95 (31.6%)	78/232 (33.6%)	0.7960
**Time from arrival to hematoma enlargement, h**				
Median	3	4	3	0.4613
IQR	2–14	3–14	2–14	
mRS 0–2 @discharge	210 (53.4%)	51 (43.6%)	159 (57.6%)	0.0113
Death within 30 days	75 (19.1%)	30 (25.6%)	45 (16.3%)	0.0356
Neurological death	56 (75.7%)	20 (71.4%)	36 (78.3%)	0.5809

*AND* allow natural death, *APTT* activated partial thromboplastin, *DI* diffuse injury, *EDH* epidural hematoma, *IPH* intraparenchymal hemorrhage, *IQR* interquartile range, *mRS* modified Rankin scale score, *PT-INR* prothrombin time (international normalized method), *SAH* subarachnoid hemorrhage, *SDH* subdural hematoma.

### Clinical outcomes

As shown in Table 1, 51 (43.6%) patients in group A and 159 (57.6%) patients in group B had mRS 0–2 at discharge (p = 0.0113). Thirty (25.6%) patients in group A and 45 (16.3%) patients in group B died within 30 days of their injuries occurring (p = 0.0356). On the other hand, the incidence of hematoma enlargement did not differ significantly between groups A and B (31.6% vs 33.6%, p = 0.7960). The incidence of neurological death also did not differ significantly between the groups (71.4% vs 78.3%, p = 0.5809). In the multivariate analyses (Table 2), the independent risk factors for poor outcome were found to be higher age (OR 32.7, p < 0.0001), female (OR 1.77, p = 0.0285), VKA use (OR 2.36, p = 0.0297), and hematoma enlargement within 72 h (OR 3.73, p < 0.0001).

**Table 2:** Results of the univariate and multivariate analysis of the risk factors associated with poor outcome in patients with tICH.

	Outcome at discharge		Univariate		Multivariate	
	mRS 0–2 (good)	mRS 3–6 (poor)	OR (95% CI)	p-value	OR (95% CI)	p-value
No. of patients	210	183				
Age, median (IQR)	69.5 (61–78.3)	78 (71–83)	32.1 (9.63–121.02)	< 0.0001	32.7 (7.62–166.30)	< 0.0001
**Sex**						
Female	56 (26.7%)	73 (39.9%)	1.83 (1.19–2.80)	0.0053	1.77 (1.06–2.98)	0.0285
Male	154 (73.3%)	110 (60.1%)				
AT, yes	51 (24.3%)	66 (36.1%)	1.76 (1.14–2.73)	0.0109		
AP	41 (19.5%)	38 (20.8%)	1.08 (0.66–1.77)	0.7595		
DAPT or TAPT	9 (4.3%)	10 (5.5%)	1.29 (0.51–3.32)	0.5874		
AC	19 (9.0%)	36 (19.7%)	2.46 (1.37–4.55)	0.0024		
VKA	11 (5.2%)	31 (16.9%)	3.69 (1.85–7.90)	0.0001	2.36 (1.09–5.39)	0.0297
DOAC	8 (3.8%)	5 (2.7%)	0.71 (0.21–2.16)	0.5491		
**Enlargement of intracranial hematoma < 72 h**						
Yes	47/204 (23.0%)	70/147 (47.6%)	3.04 (1.93–4.83)	< 0.0001	3.73 (2.25–6.28)	< 0.0001
**Extracranial injury**						
Yes	51 (24.3%)	46 (25.1%)	1.05 (0.66–1.66)	0.8453		

*AC* anticoagulant agents, *AP* antiplatelet agents, *AT* antithrombotic agents, *DAPT* dual antiplatelet therapy, *DOAC* direct oral anticoagulants, *IQR* interquartile range, *mRS* modified Rankin scale score, *OR* odds ratio, *TAPT* triple antiplatelet therapy, *VKA* vitamin K antagonist.

Sub-analyses revealed that hematoma enlargement occurred in 21 of 44 (47.7%) patients that were being treated with AC and in 87 of 283 (30.7%) patients that were not being treated with such drugs. Hematoma enlargement also occurred in 80 of 146 (54.8%) patients in whom IPH was detected on the 1st CT scan and in 28 of 181 (15.5%) patients in which IPH was not detected on the 1st CT scan. AC use and the presence of IPH on the 1st CT exhibited significant associations with hematoma enlargement (p = 0.0376 and p < 0.0001, respectively). The use of AP, however, was not associated with hematoma enlargement (22.7% vs 35.6%, p = 0.0565).

The duration of the period from arrival to hematoma enlargement did not differ significantly between groups A and B (4 vs. 3 h, p = 0.4613). In almost all cases, hematoma enlargement occurred within 24 h, regardless of whether the patient was taking AT. Only 1 patient without AT was detected hematoma enlargement after 50 h from onset because 2nd CT was not performed until then.

### Reversal agent for AC

In 55 patients of AC user, 9 patients received 4F-PCC in addition to Vit. K2, 20 patients received Vit. K2 only, and 1 patient received Idarucizumab as a reversal agent for AC. The median INR on arrival was 1.7 (IQR 1.3–2.3) in patients without reversal agent, 2.3 (IQR 2.1–2.5) in patients with 4F-PCC, and 2.4 (IQR 1.7–3.4) in patients with Vit.K2 only. There was significant difference in reversal agent users and in non-users (2.3 vs 1.7, p = 0.0095), and there was no significant difference in patients with 4F-PCC and in patients with Vit. K2 only (p = 0.9436). Hematoma expansion occurred in 7 of 20 patients (35.0%) who were not used reversal agents, in 3 of 9 patients (33.3%) reversed by 4F-PCC, and in 13 of 20 patients (65%) reversed by Vit. K2 only. Hematoma expansion tend to be occurred less frequently in patients with 4F-PCC than in patients with Vit. K2 only, although there was no significant difference (p = 0.2256).

### Thromboembolic events

**Figure 2:**
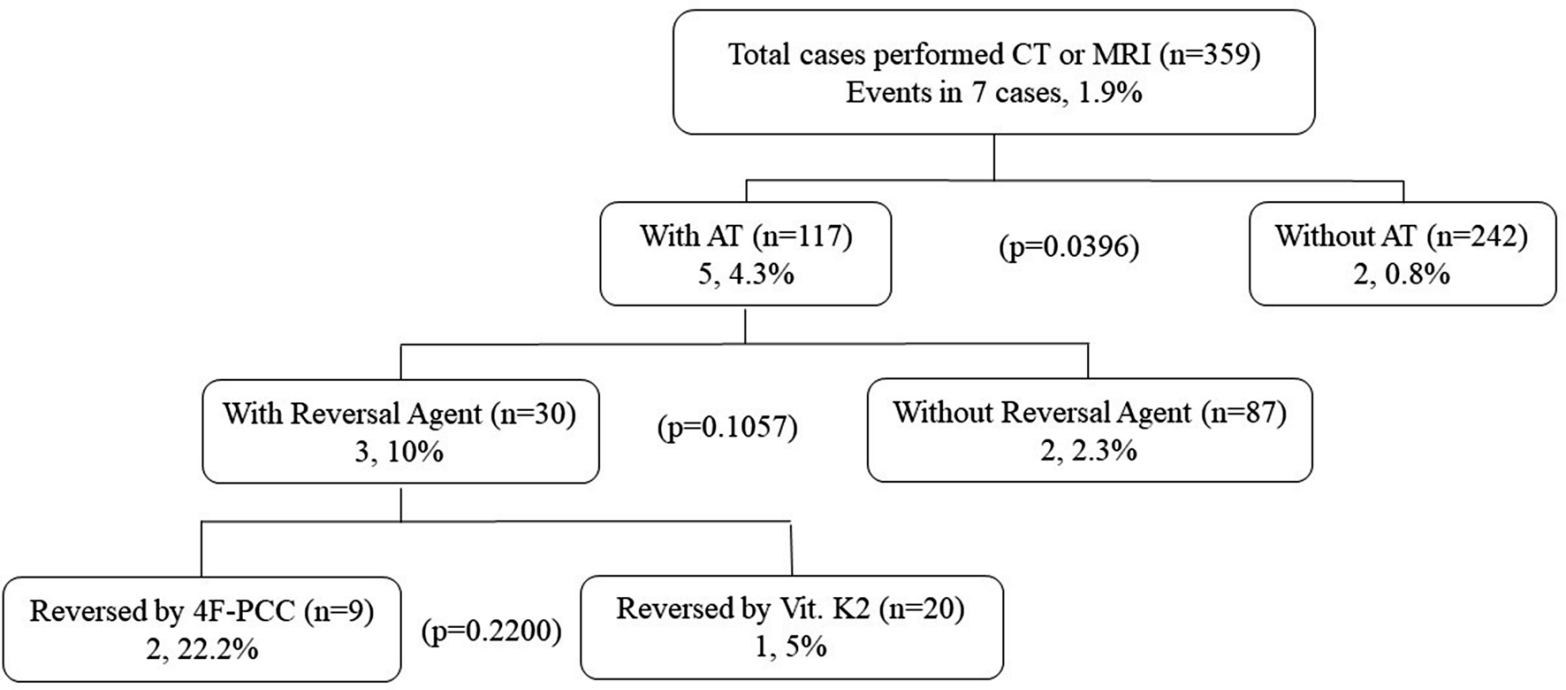
The incidence of ischemic stroke in patients with traumatic intracranial hemorrhage. There was significant difference in the incidence of ischemic stroke between AT users and AT non-users. And ischemic stroke tend to be occurred in reversal agent users, especially in 4F-PCC users.

Repeated CT or MRI were performed in all patients of group A and 242 patients (87.7%) of group B. There were totally 7 patients of ischemic stroke after admission (Fig. 2) and no patients of MI, PE or VTE in this study. Ischemic stroke occurred in 5 patients (4.3%) of group A and in 2 patients (0.8%) of group B, and incidence of ischemic stroke was significantly higher in group A (p = 0.0396). In group A, 3 of 30 patients (10%) with reversal agent and 2 of 87 patients (2.3%) without it had ischemic stroke. Ischemic stroke tended to be occurred more frequently in patients with reversal agent than in patients without it, although there was no significant difference (p = 0.1057). Furthermore, ischemic stroke was occurred in 2 of 9 patients (22.2%) reversed by 4F-PCC and in 1 of 20 patients reversed by only Vit. K2. Ischemic stroke tend to be more frequent in patients used 4F-PCC. In group A, all ischemic strokes occurred by day 31 after the injury. Three of 5 patients in group A suffered ischemic stroke before AT was restarted. In a patient used 4F-PCC, an ischemic stroke occurred on day 1.

### Rebleeding after resumption of AT

AP was restarted in 21 of 79 patients (26.6%), and AC was restarted in 35 of 55 patients (63.6%). The median days from admission to resumption was 6 (IQR 3–11) days. Rebleeding occurred in 2 cases in which AC was restarted within 2 days of the injury, and these events made the patients’ outcomes worse. There were no cases of rebleeding in AP users and in AT non-users.

## Discussion

This study investigated the clinical characteristics and outcomes of head trauma patients with intracranial hemorrhage that were taking AT. The features of clinical characteristics of AT users were lower frequencies of IPH and extracranial injuries compared with the patients that were not taking AT. In case of high-energy trauma, IPH and extracranial injury may increase. These clinical features suggest that tICH was occurred more frequently in AT users than in non-users even in cases of low-energy trauma, which is consistent with past literature^6^. The comparison between two groups showed that pre-injury AT was associated with unfavorable outcome. Furthermore, multivariate analysis revealed that VKA use, as well as higher age, female gender, and hematoma expansion within 72 h, but not AP or DOAC use, were associated with unfavorable outcome.

Pre-injury use of AC, especially VKA, was reported to be associated with increase mortality following head trauma^6–9^. A retrospective cohort study revealed that the adjusted OR for 30-day mortality of VKA users was 8.3 (95% CI 2.0–34.8)^9^. Another retrospective study also revealed that VKA was a significant predictor of the progression of IPH after head trauma compared with non-use of VKA (OR 5.30, 95% CI 2.48–11.31)^6^. In contrast, the influence of pre-injury use of AP for outcome is conflicting. A meta-analysis indicated that patients with AP had a higher risk of IPH and hematoma expansion compared with patients without AP after mild head trauma^10^. A multicenter retrospective observational study also revealed that AP was a risk factor for unfavorable outcome in patients with head trauma^7^. They reported that the risk of hematoma enlargement in the patients using AP was two-fold higher and the risk of a poor outcome was 50% higher (relative risk 1.58, 95% CI 1.28–1.95)^7^. On the other hand, a large cohort study showed that no increased hematoma enlargement in patients with AP and no increased mortality risk in patients with SAPT^6^. These differences of influence of AP for outcome and hematoma enlargement after head trauma might be caused by biases of clinical characteristics. The results of this study suggest that the influence of AP on outcome and hematoma enlargement is smaller than that of VKA.

In this study, almost all cases of hematoma enlargement detected within 24 h after the initial injury, regardless of whether the patients were using AT. On the other hand, the time from the injury to hemostasis being achieved could not be examined. Two retrospective study of spontaneous IPH reported that most hematoma enlargement occurred within 24 h from onset^11,12^. In contrast, a prospective cohort study of spontaneous IPH with VKA revealed that VKA users were at risk for bleeding well beyond 24 h^13^. To our knowledge, there are no reports that investigated the timing of hemostasis after head injury with AT. In this study, pre-injury AC was associated with hematoma enlargement within 72 h. In addition, pre-injury VKA and hematoma enlargement were associated with unfavorable outcome. Scotti et al. revealed that the patients with DOAC had substantially lower rates of IPH progression compared with the patients with VKA^6^. From these results, it is speculated that aggressive treatment by reversal agents for pre-injury VKA is important.

Two randomized controlled trials of 4F-PCC vs. Vit. K2 or plasma for cases of spontaneous IPH involving patients that were taking VKA showed that 4F-PCC had a superior ability to rapidly reduce the INR^4,14^. In addition, 4F-PCC reduced the frequencies of hematoma enlargement and hematoma-related early death in spontaneous IPH patients^15^. In this study, although there were no significant differences, the hematoma expansion rate was halved in the patients administered 4F-PCC compared with the patients reversed by Vit.K2 only. The use of 4F-PCC might thus be effective to reduce hematoma expansion after head trauma.

As the number of patients treated with reversal agents has increased, on the other hand, the frequency of thromboembolic events may increase. In this study, ischemic strokes occurred in 2 of the 9 patients (22.2%) in which 4F-PCC was used and this rate was relatively higher than in patients using Vit. K2 only (5%). To prevent thromboembolic events, the early resumption of AC might be necessary. In a retrospective study of chronic subdural hematomas, early resumption of AT was recommended because many thromboembolic events occurred within one month of drug withdrawal^16^. In a meta-analysis of spontaneous IPH, the resumption of AC between days 10 and 39 after onset produced good results^2^. In this study, there were 2 cases of rebleeding after AC was restarted within 2 days of admission. And these event influenced their outcomes. To determine the timing of resumption of AT for tICH cases, we need to collect more cases.

### Limitations of this study

This study was retrospective, and hence, background factor variability was its greatest limitation. As these differences of background likely influenced the treatment outcome, multivariate analysis was performed to reduce the bias. Nevertheless, because this was a retrospective study, biases could not be eliminated. In addition, an important limitation to the present study is the potential for underestimation of hematoma enlargement. Hematoma formation mainly occurred during initial a few hours from injury, so the detection rate of hematoma enlargement depend on the duration from injury to first CT scan. In this study, however, the time from injury to the first CT scan was not evaluated because of data missing. As a result, the amount of hematoma enlargement might be underestimated. Furthermore, treatment for head trauma, the timing of repeat CT or MRI, and whether additional CT scans were performed or not were decided at the discretion of the doctor in charge. These differences might be affected the outcomes, the rate of ischemic stroke and the rate of rebleeding. Furthermore, it was difficult to classify ischemic stroke, which occurred by thromboembolism or direct injury. Because there were small number of cases, the efficacy of reversal agents, resumption of AT, and the timing of resumption are still controversial. A prospective cohort study is needed to provide more accurate data about the outcomes of head trauma among patients taking AT.

In conclusion, patients that are taking AT are at high risk of a poor prognosis after head trauma, even after low-energy head trauma. The prognosis of those that exhibit pre-injury VKA and acute hematoma enlargement are poor, and active hemostasis, such as using reversal agents, could improve the outcomes.

## Data Availability

The datasets generated during and/or analysed during the current study are available from the corresponding author on reasonable request.
